# Interpopulation Plasticity in a Darkling Beetle Life-History along a Whole Oceanic Island Altitudinal Gradient

**DOI:** 10.3390/insects12121137

**Published:** 2021-12-19

**Authors:** Heriberto López, Sandra Hervías-Parejo, Elena Morales, Salvador De La Cruz, Manuel Nogales

**Affiliations:** 1 Island Ecology and Evolution Research Group, Instituto de Productos Naturales y Agrobiología (CSIC-IPNA), 38206 La Laguna, Spain; mnogales@ipna.csic.es; 2 Oceanography and Global Change Department, Institut Mediterrani d’Estudis Avançats IMEDEA (CSIC-UIB), 07190 Esporles, Spain; shparejo@gmail.com; 3 Grupo de Investigaciones Entomológicas de Tenerife (GIET), Departamento de Biología Animal, Edafología y Geología, Universidad de La Laguna, 38206 La Laguna, Spain; operador1.biota@gmail.com (E.M.); delacruzlopez@gmail.com (S.D.L.C.)

**Keywords:** body size, Canary Islands, *Pimelia laevigata* *costipennis*, population density, reproduction strategies, Tenebrionidae

## Abstract

**Simple Summary:**

Young oceanic islands often harbour poor biodiversity because they need time to be colonised. However, some animal species can be abundant, presenting high densities and being widely distributed through different altitudinal habitats of high oceanic islands. This is the case of the darkling beetle *Pimelia laevigata costipennis* Wollaston, 1864 on El Hierro, the westernmost island of the Canary archipelago. Therefore, this kind of framework is ideal for studying the variation of one insect species in body size, reproductive strategies, and populations in different environments. This study shows how environmental factors related to rainfall and temperature affect the different populations of this flightless beetle. Furthermore, in the more favourable habitats, located at higher altitudes, these beetles were larger in body size, laid clutches with smaller eggs, the reproduction occurred later, and showed the highest population in abundance. Lastly, this is the first study that assesses the changes of an insect along a whole insular altitudinal gradient, demonstrating apparent modifications to each habitat condition.

**Abstract:**

Insects show remarkable phenotypic plasticity in response to changing environmental conditions. The abiotic factors that determine their phenotypes often vary in time and space, and oceanic islands harbour ideal environments for testing predictions on this matter. The ubiquitous beetle *Pimelia laevigata costipennis* Wollaston, 1864 (Tenebrionidae) is distributed over the entire altitudinal gradient of the island El Hierro (Canary archipelago), from 0 to 1501 m above sea level. Here, we examine how environmental factors (i.e., rainfall and temperature), associated with the altitudinal gradient, affect the body size, reproductive phenology, clutch size and egg volume, and population dynamics of this ectothermic flightless insect. *Pimelia l. costipennis* populations inhabiting upland localities, typified by lower temperatures, and greater precipitation and vegetation cover, were larger in body size and laid larger clutches with smaller eggs than those in the lowlands. Moreover, reproduction occurred earlier in the year at lower sites and later at higher sites, whereas activity density was highest in the uplands where it increases with temperature. This study first explores the changes in life history patterns along a whole insular altitudinal gradient, and finds interpopulation plasticity. It confirms that environmental factors associated with species spatial distribution act additively as drivers of phenological and phenotypic expression.

## 1. Introduction

The capacity of a single genotype to exhibit variable phenotypes in response to changing environmental conditions (i.e., phenotypic plasticity) is typical in insects (see review in [1]). For instance, the divergence of body size, mating and life-history strategies, population dynamics, alternative morphologies, and diapause are stimulated by plastic responses to adverse factors such as low temperature or poor nutrition [2,3,4,5,6,7,8]. Altitudinal ranges are generally accompanied by heterogeneous environments including microclimatic gradients [9,10]. Species distributed over such varied habitats have a certain phenological and phenotypic plasticity to adapt without compromising their fitness or biological success [11,12]. Ecological and evolutionary responses to abiotic change across spatial and temporal scales require advances in understanding. These will improve our capacity to predict how species will respond to local and global environmental change [13,14].

Geographical variation in body size is often manifested as altitudinal or latitudinal clines (Bergmann’s rule) in response to temperature and season length [15,16,17,18]. Both increased and decreased body sizes with higher altitude and/or temperature have been noted in ectotherms. Increases in body size with altitude are explained by a negative correlation between developmental temperature and size in unlimited resource environments [4,8,19,20]. On the other hand, reductions in body size with altitude are attributed to resource limitations that restrict potential growth [21,22].

Insects also show variations in egg and clutch size and activity periods over altitudinal gradients. Egg and clutch size tend to increase in less competitive environments or longer seasons at high altitudes [23,24,25,26]. In contrast, the association between activity density and altitude is not so clear, since declines, increases, and even no altitudinal trends are found in abundance, but preferences for a particular habitat (e.g., vegetation diversity) have been documented [27]. These observations may, however, reflect only fragments of current patterns, since they concern a small proportion of insect diversity and the species involved are rarely studied over their entire altitudinal range [28]. Moreover, studies of phenotypic plasticity have traditionally focussed on analysing single isolated environmental factors (often temperature). However, temperature variation with altitude is generally accompanied by other environmental variables (e.g., precipitation, habitat type, vegetation cover) which may act additively or nonadditively on phenotypic expression [14].

Oceanic islands are ideal environments for testing the predictions of phenotypic changes with altitude because of the high degree of environmental variation and unpredictability over small spatial scales. *Pimelia* Fabricius, 1775 (Coleoptera; Tenebrionidae), a flightless darkling beetle genus inhabiting the Canary Islands (East Atlantic Ocean), provides an excellent general model to test how environmental factors promote phenological and phenotypic expression. This is due to the abundance of its species and how they are distributed in these islands. *Pimelia* has 13 endemic taxa in this archipelago [29], all belonging to the subgenus *Aphanaspis* Wollaston, 1864 [30], and several of them are distributed throughout all the habitats of their islands [31]. This is the case of *Pimelia laevigata* Brullé, 1838, a species distributed over the three westernmost islands, with a subspecies on each of them: *P. l. validipes* Wollaston, 1864 on La Gomera, *P. l. laevigata* Brullé, 1838 on La Palma, and *P. l. costipennis* Wollaston, 1864 on El Hierro. These three subspecies have a wider range of distribution on their respective islands, with a considerable density of individuals from the lowest to the highest altitudes. They occupy a range of habitats and are generally affected by the different environmental conditions in each.

Despite the Canarian *Pimelia* being a useful model for studies in evolutionary ecology, most publications so far deal with their systematics and taxonomy [31,32,33,34] or phylogenetic relationships [35,36,37,38]. Only a few studies have examined *Pimelia* species distributed in narrow altitudinal bands, to assess their plastic or evolutionary responses to specific local environmental conditions [39,40,41], but not over wide altitudinal gradients. In this study, we focus on whether there is a variation in the body size of *P. l.* *costipennis* across its distribution over the entire altitudinal gradient of El Hierro, and if environmental factors (temperature, precipitation, habitat type, and vegetation cover) influence the size of the eggs and clutches laid, the reproductive phenology, and the population dynamics of this species.

## 2. Materials and Methods

### 2.1. Study Area and Species

**Figure 1:**
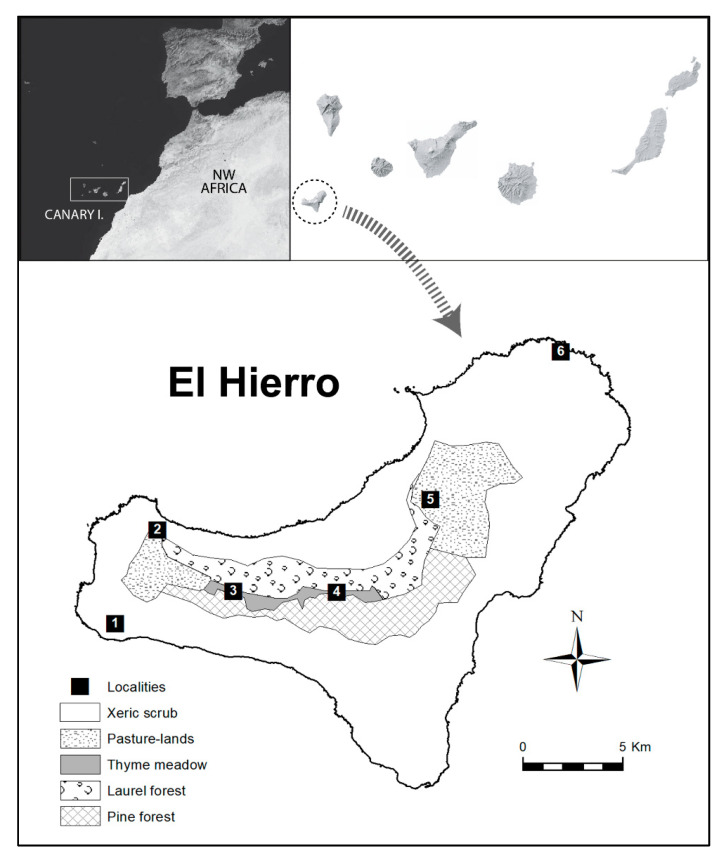
The geographical location of the island of El Hierro with its main present habitats and the six study localities: (1) Orchilla; (2) Bascos; (3) Binto; (4) Malpaso; (5) Jinama; (6) Charco Manso.

El Hierro (≈1.1 ma; [42]) is the smallest (287 km^2^) and south-westernmost (27°37′–29°25′ N and 13°20′–29°25′ W) of the Canary Islands, excluding islets. Its climate is typically arid Mediterranean (but oceanic) with a mean yearly temperature around 18 °C and annual rainfall of 350 mm. Four predominant natural habitats can be identified on the island (Figure 1): dry scrublands (up to 300–350 m a.s.l. (above sea level)), thermosclerophyllous woodland (300–600 on south-facing slopes, 200–350 m a.s.l. on north-facing slopes), laurel forest (mainly on north-facing slopes from the limit of thermosclerophyllous woodland to the summit of the island), and pine forest (mainly on south-facing slopes from the limit of thermosclerophyllous woodland to the summit of the island) [43]. However, most of the habitats were felled or cleared and transformed in the past to develop agriculture and farming [44]. An important part of the territory occupied in the past by laurel forest is now a characteristic landscape unit constituted by wide grazing meadows. In turn, pine woodland at the highest elevations become more open with sparser trees, with the ground covered largely by Canary thyme meadows of *Micromeria hierrensis*.

**Figure 2:**
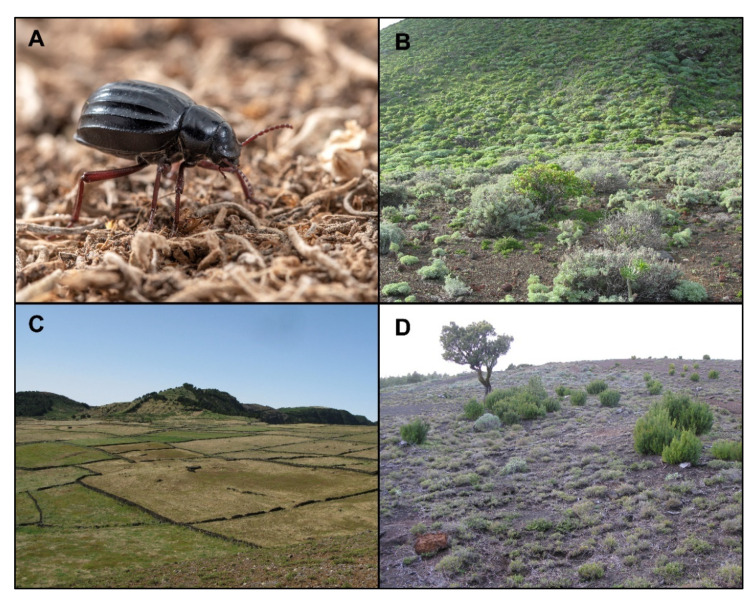
Aspect of *Pimelia laevigata costipennis* (Photo: M. Arechavaleta) (**A**), and aspect of the habitat’s dry scrublands (DS) at low altitudes (**B**), pasture and crop land (PL) at medium altitudes (**C**), and Summit Canary thyme meadows (TM) of *Micromeria hierrensis* at high altitudes (**D**).

To carry out this study we have selected *Pimelia l. costipennis* (Figure 2A), a species distributed over the entire altitude gradient of the island, from 0 to 1501 m above sea level. The populations living at higher altitudes face lower temperatures, more abundant precipitation, greater vegetation cover, and longer wet seasons than those at lower altitudes. Its populations become so abundant in certain parts of the island that some seabirds include it in their diet, altering their normal feeding behaviour [45]. This *Pimelia* seems to adapt well to any habitat and shows polyphagous habits, although with clear preferences for tender green parts of herbaceous vegetation (H.L., pers. obs.). Furthermore, after several years of fieldwork on the island, visiting all these habitats in the frame of this and other studies, we have found its highest population densities in open habitats. It is much scarcer in dense wooded areas with little herbaceous ground cover. For this reason, in this study we avoided sampling in the dense forest, to obtain suitable sample sizes for the proposed analysis.

Fieldwork was conducted over an entire year (from January 2012 to January 2013) along the whole island altitudinal gradient (Figure S1), and in the three habitats where we verified that *P. l.* *costipennis* was especially abundant (see Figure 1): (i) dry scrublands (DS) at low altitudes (up to 350–400 m a.s.l.) (Figure 2B); (ii) crop and pasture lands (PL) at mid-altitudes (mainly 400–1240 m a.s.l.) (Figure 2C), that currently replace potential laurel forest vegetation; and (iii) Canary thyme meadows (TM) in open pine forests at high altitudes (mostly above 1240 m a.s.l.) (Figure 2D).

A thermometer and a rain gauge were installed at each locality to record monthly minimum and maximum absolute temperatures and total precipitation. We performed 15 line-intercept transects 20 m long at each locality to assess the mean vegetation height and coverage. Percentage cover per species was estimated by measuring the length of the line touching each plant on the ground or rocks, following the method described in Kent and Coker [46]. In addition, we noted individual plants’ height and their positions along the previously laid measuring tape (Table 1).

### 2.2. Activity Density (AD)

At each of these six localities, two separate plots with similar soil, wind exposure, and slope were selected to assess the annual activity density (AD) of *P. l.* *costipennis* using pitfall traps. This technique can be used to estimate the activity density for ground-active species or single species [47,48,49,50,51]. A rectangular grid with three lines of 10 traps each was established in each plot. Lines were separated by about 10 m and traps by 5 m, providing 0.18 Ha of total sampled area at each locality. Traps were composed of a pair of plastic cups (diameter 9 cm, depth 12 cm), one inside the other, with small perforations in the bottom for any rainwater to drain away. The outer cup acted as a permanent hole allowing the inner one to be installed flush with the soil surface and be easily removable. In addition, the set trap was protected with a large stone to impede the entrance of rainwater, excess sunlight, or predators. Traps were set for six days each month without baits or preservative liquid. After this period, the internal cups were withdrawn and the number of *P. l. costipennis* was recorded before their release at the same place. The inner cups were then replaced and covered with soil to prevent any animals entering until the following sampling dates. Females, captured to estimate the monthly ovarian maturation, as explained in the previous section, were collected at least 300 m away from these plots to avoid possible bias affecting the AD study area (distance selected based on our previous experience).

For each locality, we estimated the monthly AD as the total number of specimens collected after six days of sampling in the two plots (60 traps in total) following Brandmayr et al. [52]: AD = [number of individuals/(number of traps × number of days)] × 10). The AD was estimated without considering their sex because it is not easily identifiable in the field. To see sexual characters, the genital valves must be opened and observed using a stereomicroscope.

### 2.3. Statistical Analysis

Changes in body size with altitude were tested using a generalised linear mixed model (GLMM) using elytra length as the response variable and adjusting data to a Poisson distribution of errors, with the R package ‘lme4’. Two factors (sex, habitat type) and one numerical variable (altitude) were included, with the interaction between ‘habitat and altitude’ as fixed factor. We considered ‘capturing site’ as a random effect in the model to account for local variation between sites.

Differences in egg volume and clutch size between the populations at different altitudes were assessed with a GLMM for each predictor variable (egg volume and number of eggs, respectively), adjusting data to a Poisson distribution of errors. We included locality, temperature, precipitation, vegetation cover, and height as fixed effects, and ‘month’ as a random effect to account for temporal variation in the study variables between localities. A Kruskal–Wallis test was used to screen the differences in temperature, precipitation, vegetation cover, and height between localities. The reproductive phenology (organised into three phases of ovarian maturation) was studied by applying categorical likelihood ratio tests.

Monthly activity density data were grouped into four seasons: winter (December, January, and February), spring (March, April, and May), summer (June, July, and August) and autumn (September, October, and November). Spatial and temporal variation in AD was assessed with a generalised linear model (GLM), adjusting data to a quasi-Poisson distribution of errors with the R package ‘stats’. This included locality, temperature, precipitation, and their interactions, and season as fixed effects.

## 3. Results

Results from the GLMM showed that body size varies with altitude between habitat types (X^2^ = 53.61; *p* < 0.001). Specifically, individuals were significantly smaller in dry scrublands in lowlands compared to crop and pasture lands at mid-altitudes (β = −0.003; *p* < 0.001) and Canary thyme meadows in the uplands (β = −0.006; *p* < 0.001). Body size also varied between sexes (X^2^ = 11.76; *p* < 0.001): females were significantly larger (elytra width: mean = 11.43 ± 0.87 SD; elytra length: 15.06 ± 1.07) than males (10.89 ± 1.02; 14.41 ± 1.32, respectively) across all altitudinal ranges (β = 0.024; *p* < 0.001).

**Figure 3:**
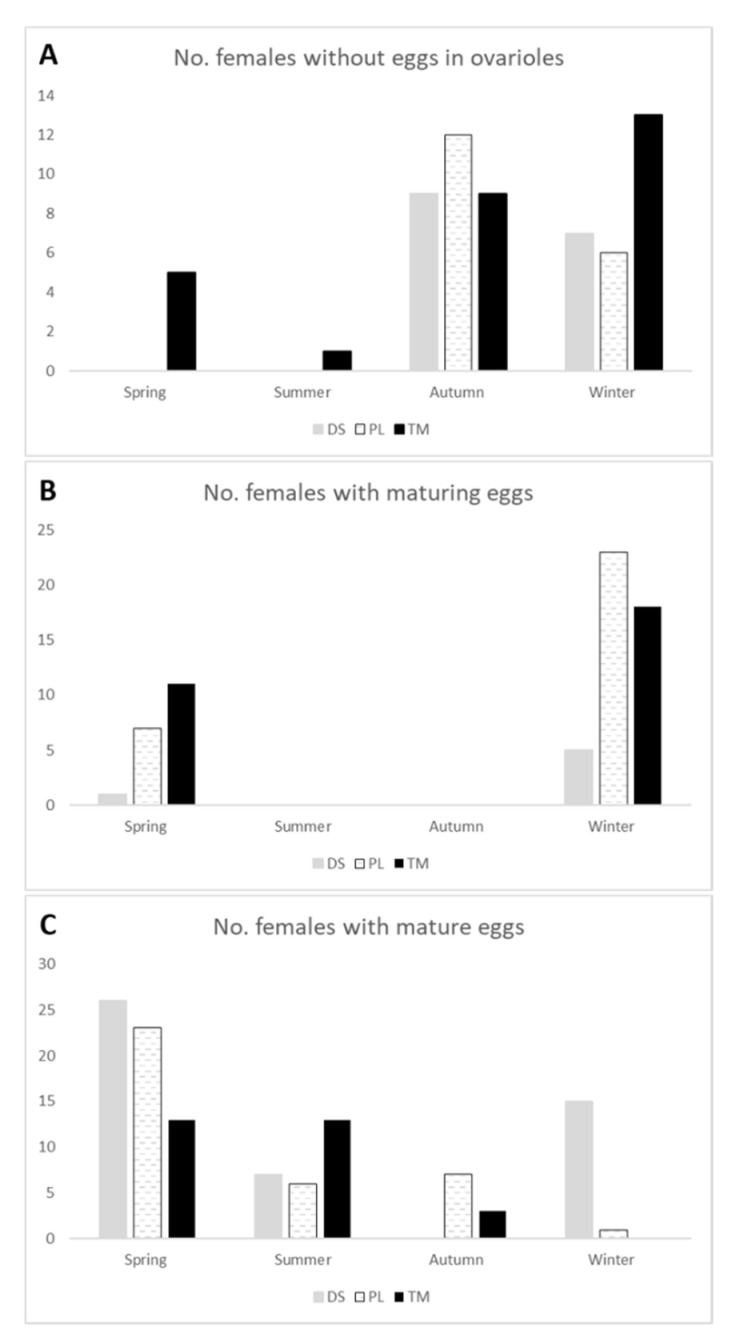
Seasonal variation of ovarian maturation of *Pimelia laevigata costipennis* in each habitat, based on dissected females. (**A**) Data on females with ovarioles without eggs; (**B**) Females with ovarioles with maturing eggs; (**C**) Females with ovarioles carrying mature eggs. DS = Dry scrublands at low altitudes; PL = Pasture and crop land at medium altitudes; TM = Summit Canary thyme meadows of *Micromeria hierrensis* at high altitudes.

Regarding the reproductive phenology of *P. l.* *costipennis*, the following was observed. (i) The early phase I (ovarioles without eggs) was significantly represented in autumn and winter in the three altitude ranges, but during the rest of the year, only females contained this type of ovarioles in the uplands (G_6_ = 13.06; *p* = 0.042). (ii) Females in phase II (ovarioles with maturing eggs) were only found in winter and spring; in the three habitats, the proportion was higher in winter but not statistically significant (*p* > 0.05). (iii) Females in phase III (ovarioles with mature eggs) were present throughout the year but the pattern varied between habitats. In dry scrublands, females had mature eggs mainly in spring and winter, in mid-altitude pastures in spring, and in uplands in spring and summer (G_6_ = 40.59; *p* < 0.001; Figure 3).

**Figure 4:**
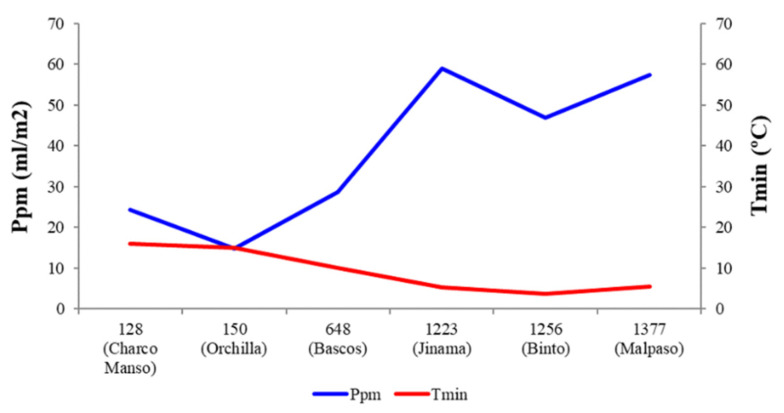
Altitudinal variation of annual mean temperature minima (Tmin) and rainfall (Ppm) at the six studied localities of El Hierro (Canary Islands). Names of localities are accompanied by their altitude (m a.s.l.).

**Figure 5:**
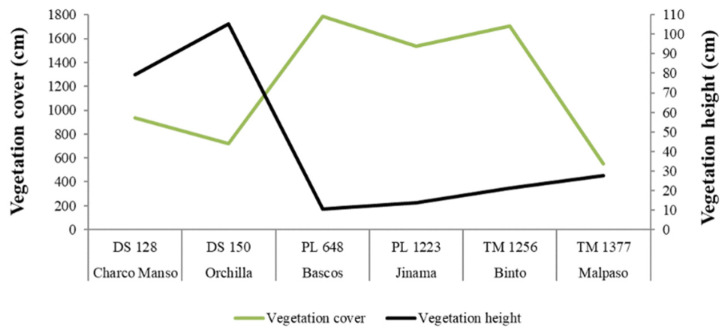
Altitudinal variation of mean vegetation cover and height at the six studied localities of El Hierro (Canaries). Habitat type and locality names are accompanied by their altitude (m a.s.l.).

Egg volume varied with temperature between localities (X^2^ = 23.00; *p* < 0.001). Eggs were larger at lowland localities with high temperatures (β = 0.037; *p* < 0.001), whereas precipitation, vegetation cover, and height had no effect on egg volume. This model, however, explained only 34.4% of the variance in egg volume. Among localities, egg clutch also varied with temperature (X^2^ = 33.92; *p* < 0.001), precipitation (X^2^ = 31.08; *p* < 0.001), and mean vegetation height (X^2^ = 30.16; *p* < 0.001). Thus, more eggs were laid by females at upland localities with low temperatures (β = 0.057; *p* < 0.001) and vegetation height (β = 0.007; *p* < 0.001), and high precipitation (β = 0.007; *p* < 0.001). The model accounted for 99% of the variance in egg clutch. A Kruskal–Wallis test confirmed the differences in temperature, precipitation, and vegetation cover and height between localities (K = 172.53; K = 109.28; K = 240.38; K = 240.59, respectively, all *p* values < 0.001). Temperature and vegetation height decreased, whereas precipitation and vegetation cover increased with altitude (Figure 4 and Figure 5). 

**Figure 6:**
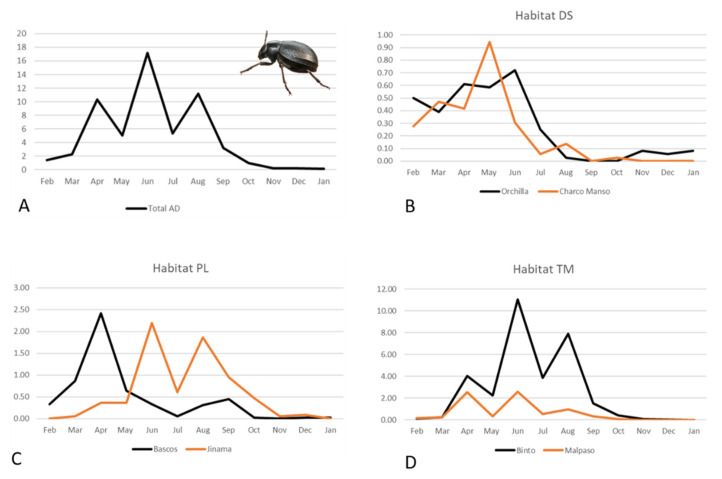
Monthly activity density (individual per trap) of *Pimelia laevigata costipennis* from El Hierro through a one-year study. (**A**) activity density at the six studied localities; the activity density of (**B**) is related to two dry scrubland localities at low altitudes (DS); (**C**) two crop and pastureland localities at mid-altitudes (PL); (**D**) two summit Canary thyme meadows of *Micromeria hierrensis* localities at high altitudes (TM).

Overall activity density of *P. l.* *costipennis* (i.e., considering the six localities together) showed three peaks: one in spring (April: 10.11 individuals per trap) and two in summer (June: 17.19 individuals per trap, and August: 11.19 individuals per trap) (Figure 6A). However, when we depict AD considering the three different habitats, a different pattern was observed. Specifically, individuals showed the lowest AD values at low altitudes and virtually no activity in autumn (Figure 6B). At mid-altitude, females displayed AD from late winter to late autumn with two peaks in April and June (Figure 6C), whereas those at high altitude recorded the highest AD values and had no activity in late autumn and winter (Figure 6D).

Results from the GLM showed that AD varied between seasons (X^2^ = 35.28; *p* < 0.001), indicating higher values in summer (β = 0.880; *p* = 0.006) and slightly lower in winter (β = −1.804; *p* = 0.049). Activity density was also positively affected by precipitation (X^2^ = 7.44; *p* = 0.006). Lastly, the interaction ‘locality*temperature’ was also significant (X^2^ = 10.74; *p* = 0.046), showing an increase in AD in upland localities as temperature increased (β = 0.400; *p* = 0.012). This model explained 78.5% of the variance in AD.

## 4. Discussion

As far as we know, this is the first study to explore the changes in life history patterns along the entire altitude gradient (from 0 to above 1200 m a.s.l.) of a native beetle species. It finds a clear phenological and phenotypic plasticity that permits it to adapt to local conditions (i.e., habitat type, temperature and precipitation, and vegetation height). These environmental variations result in divergence between populations in terms of body size, phenology of reproduction, egg and clutch size, and activity density along the altitudinal gradient.

### 4.1. Altitudinal Plasticity in Body Size

*Pimelia l. costipennis* populations inhabiting upland localities had a larger body size compared to those in the lowlands (coinciding to Bergmann’s rule). This pattern may be due to the differences in the environmental conditions across the altitudinal gradient. Indeed, individuals inhabiting higher altitudes face lower temperatures and more abundant precipitation than those at lower altitudes. Moreover, the habitat type could also play an essential role in explaining body size variation with altitude. This pattern could be related to the increase in vegetation cover with altitude, thus providing thermal shelters for darkling beetles at cold temperatures [53].

Previous studies showed that Tenebrionidae distributed on the European mainland are in line with a positive temperature–size relationship [54,55]. However, our findings seem consistent with a negative ‘temperature–size rule’ in ectotherms, as do other authors [19,56,57,58]. Various mechanistic drivers of this rule have been proposed. One is that temperature changes the growth/reproduction trade-off [59], and another is that increasing metabolic demand due to temperature is balanced by a constant or decreasing environmental supply [60]. However, recent studies suggest that this pattern may be multicausal, driven by many ecological and acclimation responses [61,62]. Therefore, more studies simultaneously considering different environmental factors are needed to foresee the effects of temperature on organism size in natural environments.

Contrary to other Tenebrionidae species (see review in [54]), precipitation also seems to favour increased body size of *P. l. costipennis* because individuals were larger at localities with high rainfall. However, in Carabidae, body size was most strongly affected by the precipitation in the driest month, suggesting that body size is small in localities with limited amounts of rain [61]. On El Hierro, frequent periods of drought in the lowlands could drive the small body size in the *Pimelia* populations, combined with other related environmental factors such as low vegetation cover.

### 4.2. Effect of Biotic and Abiotic Factors on the Phenology of Reproduction, Eggs and Clutch Sizes

The greater volume of rainfall at medium and high altitudes on El Hierro, together with moderate temperatures during the summer (see Figure S2), contributes to these habitats providing fresh vegetation for longer periods. This pattern could explain why *P. l. costipennis* females extend their reproductive cycle at middle and high altitudes, containing inside mature eggs even after summer, as in other species (see [63]). On the other hand, lower down, where rainfall is lower, and temperature rises considerably after winter (see Figure S2), the vegetation dries out earlier in the year. So, the availability of fresh food will only be restricted to winter months. Under these limited ecological conditions, the additional energy required to produce mature eggs probably leads to females shortening their reproductive cycles, which are restricted to the winter months in the xeric lowlands of El Hierro.

There is much less literature about plasticity in ovarian maturation than that in body size. Previous authors have pointed out the difficulty to predict the magnitude or direction of the effect of temperature on offspring size without considering clutch size [64,65]. Here, we quantified a negative relationship, or trade-off, between egg size and clutch size that could be explained, for example, by a decrease in the availability of resources (i.e., fresh vegetation) due to higher temperature. Previous studies show that a reduction in food availability or the predominance of higher temperatures can affect the fecundity of eggs or increase progeny mortality (see review in [66]). Under these environmental conditions, females can shift to laying larger eggs (see [67]). Offspring that hatch from larger eggs are open to greater survivorship because larvae have more nutrients, probably hatch with greater size, and can better overcome environmental adversities [68,69].

The egg-size response to temperature found here is the opposite of that observed in most species (see review in [66]). Larger eggs can be adaptive in colder environments due to a faster postembryonic development rate, which is advantageous if the laying season shortens [70,71]. However, large egg size also confers greater tolerance to high temperatures [72], as presumably happens in the lowlands of El Hierro (see temperatures in Table 1).

In our study, upland clutch size seems to associate positively with female body size, since upland females and their clutches were both larger there. The positive effect of precipitation on clutch size has been described in skinks, for example, due to extreme lowland conditions constraining them to laying multiple small clutches [73]. However, the effect of precipitation on the clutch size of beetles deserves further attention.

### 4.3. Environmental Drivers of Activity Density

Although increased dryness and aridity could affect populations of coleopterans negatively, many darkling beetle species exhibit their highest AD in the period of the highest temperature and the lowest precipitation (summer) (see [74,75,76,77]). These two latter climatic factors were also important drivers of life cycle variations in *P. l. costipennis*, considering its entire altitudinal distribution on El Hierro. Previous studies in the Canary Islands showed that one species of *Pimelia* from the lowlands of the larger island Tenerife increased in AD when the temperature decreased. In contrast, another upland species increased its AD with temperature [40]. Our study supports these previous findings, but for an entire altitudinal distribution gradient of the same species. Therefore, this adaptation of AD in *Pimelia* appears to be related to adaptation mechanisms to environmental conditions associated with altitude and not inherent to this or any other species. In this case, the activity period was higher at high altitudes where *P. l. costipennis* considerably increased its AD, up to nine times more compared to the lowlands. Activity periods of *P. l. costipennis* based on both suitable thermal regimes and humidity might explain the high AD in the uplands.

### 4.4. Concluding Remarks

Our study confirms that several abiotic factors (i.e., temperature, precipitation) and vegetation height associated with altitude act additively on *P. l. costipennis* as drivers of phenological and phenotypic expression. It also highlights the importance of considering the whole altitudinal gradient to disentangle interpopulation plasticity. Overall, we conclude that the causes of variation in the body size, reproductive strategies, and the life cycle of this darkling beetle are complex and being driven by multiple environmental and ecological factors. Further insights from studies on food supply and energy demands would aid towards understanding the ecological strategies that species develop to adapt their life history under different climatic influences, especially in the current scenario of global change.

## Figures and Tables

**Table 1:** Environmental factors in the study areas. DS = dry scrublands; PL = pasture lands and crop fields; TM = Summit thyme meadows of *Micromeria hierrensis*.

Locality	Habitat Type	Altitude (m a.s.l.)	Annual Rainfall (mL/m^2^)	Mean Tmin (°C) (Range)	Mean Tmax (°C) (Range)
**East region**					
Charco Manso	DS	128	240	15.9 (10–21)	32.2 (28–37.5)
Jinama	PL	1223	707	5.3 (1–11)	25.7 (10–38)
Malpaso	TM	1377	690	5.4 (1–10.5)	29.1 (22–38)
**West region**					
Orchilla	DS	150	177	15.1 (10–24.5)	33.3 (26–43)
Bascos	PL	648	344	10.1 (4.5–14.5)	27.6 (21–37.5)
Binto	TM	1256	563	9.1 (−1.5–7.5)	33.3 (19.5–36.5)

## Data Availability

All data are available from the authors upon request.

## Supplementary Materials

The following are available online at https://www.mdpi.com/article/10.3390/insects12121137/s1, Figure S1: *Pimelia l. costipennis* capture sites; Figure S2: Climate graphs of the six studied localities.
