# Supplementary material for: Interpopulation Plasticity in a Darkling Beetle Life-History along a Whole Oceanic Island Altitudinal Gradient
Source: Insects. 2021 Dec 19;12(12):1137. doi: 10.3390/insects12121137 (PMC8707004; doi:10.3390/insects12121137)
Supplement: Supplementary file 1 [file insects-12-01137-s001.zip › insects-1497096-supplementary.pdf]

insects

MDPI

Supplementary Material

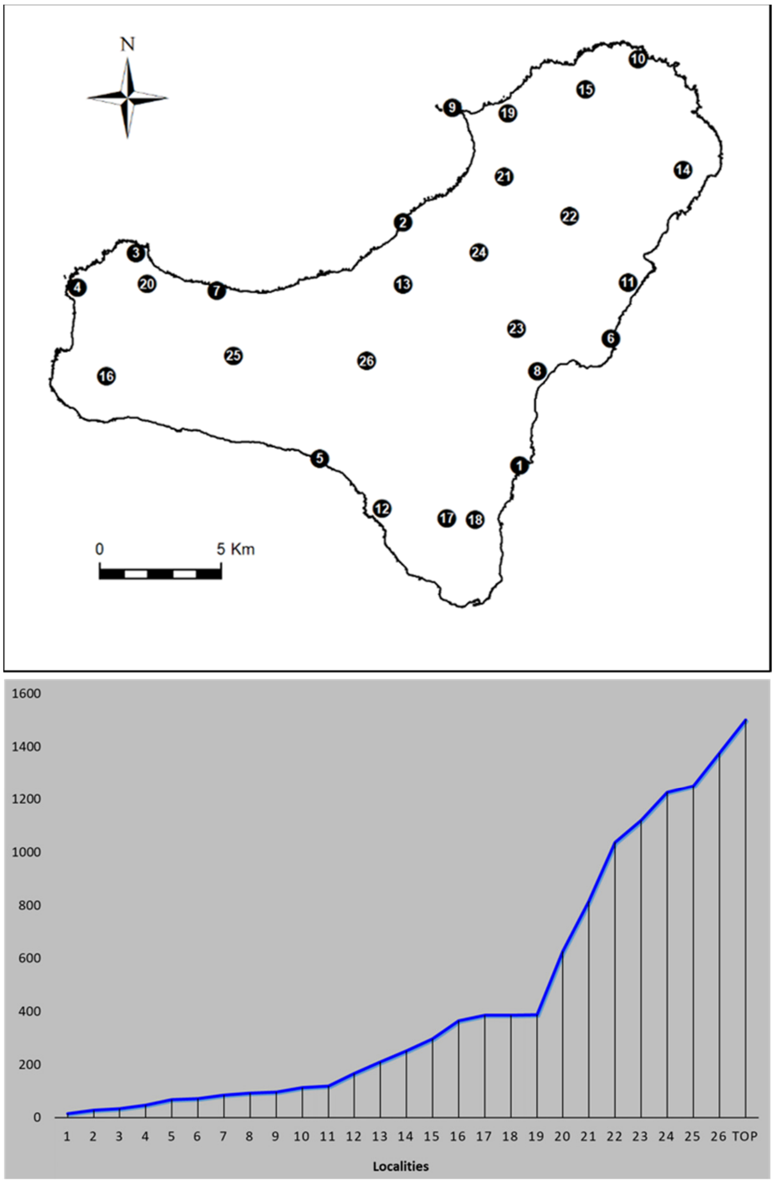

Figure S1. Geographical location of the 26 *Pimelia l. costipennis* capture sites. Individuals were collected to determine their body size throughout the entire altitudinal gradient of El Hierro. The site TOP in the graph is the maximum altitude of the island (1501 m a.s.l.).

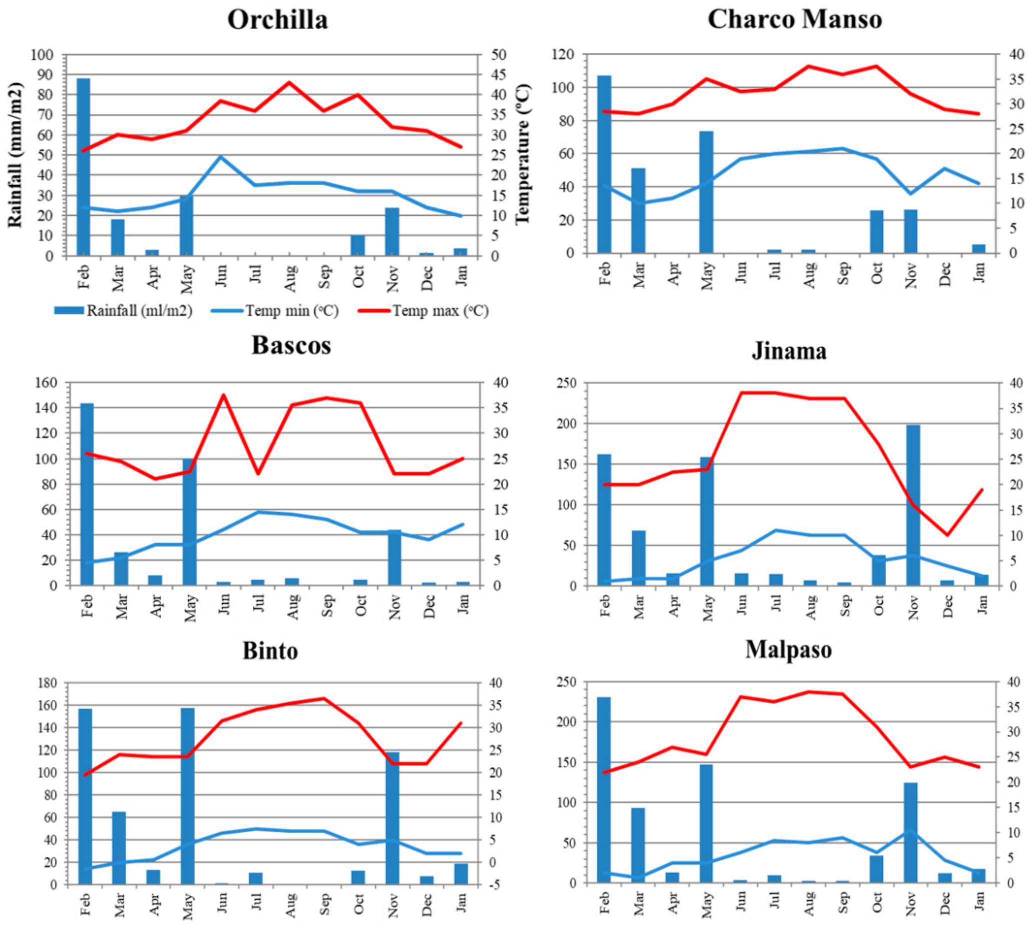

Figure S2. Climate graphs of the six studied localities on El Hierro: Orchilla and Charco Manso (dry scrublands at low altitudes), Bascos and Jinama (pasture lands at mid-altitudes), and Binto and Malpaso (thyme meadows at high altitudes).
